# Supplementary material for: Behavioral assessment of children and adolescents with Graves’ disease: A prospective study
Source: PLoS One. 2021 Apr 29;16(4):e0248937. doi: 10.1371/journal.pone.0248937 (PMC8084231; doi:10.1371/journal.pone.0248937)
Supplement: Attachment — Submitted filename: Response to reviewers comments R3.docx [file pone.0248937.s001.docx]

We are grateful to the valuable and helpful reviewers' and editor's comments on our manuscript ID # [PONE-D-20-38682R2] with the title "**Behavioral assessment of children and adolescents with Graves' disease: A prospective study**" submitted as an original article wishing to be published in Plos One. We have addressed all comments (point-by-point), as indicated in the attached pages and considered their corrections within the main text. We hope that the explanations and revisions of our work are satisfactory. We have highlighted the changes in the revised manuscript (yellow color).

**Response to reviewer's # 1 comments**

**Reviewer:**

1. The manuscript is improved. Most of the problems which remain are related to language rather than to scientific issues.

**Author:**

The text of manuscript has been edited by a colleague who naive language is English.

**Reviewer:**

2. Abstract Line 7: Instead of 'active and normalized thyroid hormone levels' states'---'during periods of thyroid hormone elevation and of normalized thyroid hormones.' Use these terms also in line 16. Similar verbiage should be used in line 4 and in the final paragraph of the introduction.

**Author:**

We have done the required corrections

**Reviewer:**

3. In 'Methods' section--line 3: omit comma between 'out-patient clinic' and and 'of Assiut University Hospital.'
   **Author:**

It has been corrected.

**Reviewer:**

4. 'Methods' section line 8: (2) those with normal thyroid hormone levels
   **Author:**

It has been corrected.

**Reviewer:**
5. 'Methods' section line 10: '...which was subsequently titrated to maintain normal thyroid hormone levels based on...'

**Author:**

It has been corrected.

With this manuscript, there are 4 tables which we wish to be published.

I certify that the materials of this work **“Behavioral assessment of children and adolescents with Graves' disease: A prospective study”** have not been submitted simultaneously elsewhere and that there is no part of the text or any of the tables has been copyrighted, published, or reproduced elsewhere. I certify that all individuals included as authors of papers have contributed substantially to the scientific process leading up to the writing of the paper and its revised version.

Thank you for accepting reviewing our manuscript.
